# The effects of neoliberal policies on access to healthcare for people with disabilities

**DOI:** 10.1186/s12939-017-0699-3

**Published:** 2017-11-15

**Authors:** Dikaios Sakellariou, Elena S. Rotarou

**Affiliations:** 1 School of Healthcare Sciences, Cardiff University, Eastgate House, Newport Road 35-43, Cardiff, CF24 0AB UK; 2 Department of Economics, University of Chile, Diagonal Paraguay 257, Office 1506, 8330015 Santiago, Chile

**Keywords:** Neoliberalism, Access to healthcare, Healthcare, Chile, Greece, Austerity, Health systems, Structural adjustment programmes

## Abstract

Neoliberal reforms lead to deep changes in healthcare systems around the world, on account of their emphasis on free market rather than the right to health. People with disabilities can be particularly disadvantaged by such reforms, due to their increased healthcare needs and lower socioeconomic status. In this article, we analyse the impacts of neoliberal reforms on access to healthcare for disabled people. This article is based on a critical analytical review of the literature and on two case studies, Chile and Greece. Chile was among the first countries to introduce neoliberal reforms in the health sector, which led to health inequalities and stratification of healthcare services. Greece is one of the most recent examples of countries that have carried out extensive changes in healthcare, which have resulted in a deterioration of the quality of healthcare services. Through a review of the policies performed in these two countries, we propose that the pathways that affect access to healthcare for disabled people include: a) *Policies directly or indirectly targeting healthcare, affecting the entire population, including disabled people*; and b) *Policies affecting socioeconomic determinants, directly or indirectly targeting disabled people, and indirectly impacting access to healthcare*. The power differentials produced through neoliberal policies that focus on economic rather than human rights indicators, can lead to a category of disempowered people, whose health needs are subordinated to the markets. The effects of this range from catastrophic out-of-pocket payments to compromised access to healthcare. Neoliberal reforms can be seen as a form of structural violence, disproportionately affecting the most vulnerable parts of the population – such as people with disabilities – and curtailing access to basic rights, such as healthcare.

## Introduction

While access to healthcare is a basic human right, according to the World Health Organisation’s Constitution, people with disabilities face several barriers in their effort to access healthcare services and report higher unmet healthcare needs, compared to people with no disabilities [1–5]. Access to healthcare is a multifaceted concept, which can refer to, among others, service availability, utilisation of services, relevance of services, and equity [6]. Concurring with previous research [7], in this article we use access to healthcare to refer to the “*opportunity to identify healthcare needs, to seek healthcare services, to reach, to obtain or use health care services and to actually have the need for services fulfilled*” (p. 8).

Following the 2008 global financial crisis, several countries implemented structural adjustment programmes that affected most economic sectors, including healthcare. However, such neoliberalism-inspired programmes are not a new phenomenon; these have been observed as far back as the early 1970s, when Chile embarked on a wide-ranging set of neoliberal reforms. Labonté and Stuckler [8] argue that such reforms – besides often having a negative impact on poverty, social cohesion, and economic growth – can have severe effects on population health, leading to health inequalities and widening socioeconomic disparities.

There is an extensive body of research on the impact of neoliberalism on access to healthcare for the general population [9–14]. However, there is a dearth of research on the effects of these policies for people with disabilities (for example, [15, 16]). Such studies have confirmed the existence of health inequalities in access and utilisation of healthcare services between people with disabilities and the general population.

Our aim in this article is to explore the interplay between neoliberal policies and access to healthcare for people with disabilities. We do this through a critical analytical review of the literature and through the use of two case studies: Chile and Greece. Guided by the World Health Organisation [17], in this article we define disability as limitations in participation in any aspect of everyday life due to the interaction between impairment or illness and the environment. This definition is inclusive of people who experience sensory or physical impairment, or who live with chronic illness, including mental illness. We use interchangeably the terms people with disabilities and disabled people throughout this article, to highlight disability as both in terms of impairment and in terms of social oppression.

Chile and Greece are high-income, Organisation of Economic Development and Cooperation (OECD) member countries that have both carried out extensive structural changes in their respective health sectors. Chile was among the first countries to introduce neoliberal reforms in the health sector, by promoting an extensive marketisation of its healthcare in the 1980s, leading to a radical restructuring of its healthcare system, and the creation and perpetuation of health inequalities in the country [14]. Greece, on the other hand, is one of the most recent examples of countries that, because of conditionality clauses attached to debt management programmes, had to carry out extensive changes in the public sector, including healthcare. Such neoliberal reforms have led to severe deterioration of the quality of the health sector and services, and therefore, have resulted in serious, negative consequences for the country’s population [18].

After offering a brief overview of neoliberalism and its effects on disabled people, we present how neoliberal policies implemented in Chile and Greece have affected access to healthcare for disabled people. We finish the article by discussing the impacts of neoliberal reforms as a type of structural violence, and we present pathways through which access to healthcare for disabled people is affected.

## Background

### Neoliberalism

The impact of neoliberalism on the economic, social, and political life has been widely investigated, particularly in the context of the structural adjustment programmes applied in the 1980s and 1990s to countries under crisis in Africa, Latin America, and the former Soviet Union. With the recent economic crisis, neoliberal policies have come again under scrutiny, as many governments – this time in Europe – have been adopting austerity measures in order to decrease their budget expenditure. Karamessini [19] states that “…*the neoliberal offensive has had a major disruptive effect on social cohesion, as well as on people’s lives and morale, especially the most vulnerable*…” (p. 176).

Neoliberalism is generally associated with a set of policies implemented in the 1980s by the International Monetary Fund, the World Bank, and the United States of America Treasury Department, in an effort to help crisis-stricken developing countries by prescribing a series of reforms, the so-called ‘Washington Consensus’ policies. Such policies aimed at achieving macroeconomic stabilisation, reducing governments’ role in the economy, privatising public assets, and reducing public expenditure [20]. While neoliberalism has acquired many economic, social, political, and philosophical definitions, it is usually associated with a general orientation towards a strongly market-based approach, which emphasises deregulation, minimalisation of the State, privatisation, and the emergence of individual responsibility [21].

For Mladenov ([22], p. 446), one “…*important element of neoliberalism is the retrenchment of the welfare dimension of the state, which is seen as an impediment to the optimal functioning of the markets*”. This retrenchment can be translated into fewer, more expensive, less controlled, and of lower quality healthcare services [23]. Furthermore, this process of reducing the welfare state moves responsibility for taking care of people from the state to the free market, leading to wide disparities in the level and quality of care people receive [14].

### Neoliberal disability

In the context of neoliberalism, people with disabilities are often, for reasons discussed later in this section, negatively evaluated [14, 24]. This is despite the fact that some of the first policies aligned with neoliberalism concerning people with disabilities were deinstitutionalisation and direct payments for managing their own care [22]. Both of these policies had also been goals of the Disabled People’s Movement (DPM) for a long time, as ways to promote autonomy, independence, and self-determination of people with disabilities [25]. As Mladenov said ([22], p. 451), neoliberalism’s “*libertarian pathos of marketisation resonated well with the emancipatory aspirations of the DPM*”.

A series of policy developments – in the areas of health and labour, mainly – have promoted a neoliberal agenda that directly affects the lives of people with disability, causing in many cases material deprivation, insecurity, and stigmatisation [26]. For example, under the pressure to reduce expenditure and welfare, new categorisations and assessment of people with disabilities have emerged aiming at redefining people’s abilities to perform paid work and receive state benefits [24]. This involves stricter eligibility criteria, and an increased pressure on disability claimants to enter employment, in an effort to re-categorise ‘disability’ and separate those who are ‘really disabled’ from those who can perform some kind of paid work [24]. Such measures indicate a neoliberal emphasis on “*work discipline, deregulated labour markets, and a flat-rate benefit welfare system providing minimum and often means-tested benefits*” ([27], p. 47). As Goodin ([28], p. 579) argued, citizenship is increasingly seen in terms of “*responsibilities and obligations*”, affecting people with disabilities around the world [24, 29, 30].

The combination of a continuing emphasis on reducing benefits and the existence of high unemployment has led many disabled people to poverty [26, 31]. In the wake of recent austerity measures, especially in the United States, Europe, and Australia, people relying on benefits have been particularly hit, as healthcare and unemployment benefits have been reduced further due to budget shortfalls [24, 30, 32]. Austerity measures coupled with efforts to re-determine what it means to be ‘really disabled’ could have repercussions regarding who is entitled to welfare support [30]. In the neoliberal discourse of abled and non-abled bodies, all bodies are evaluated in terms of their success of failure in achieving health, wealth, or career realisation.

In this climate, we can observe the emergence of neoliberal disability: disabled people are often viewed as *costly bodies* who use up limited healthcare resources [15] or as potentially *financially burdensome* [24]. This negative evaluation can be further exacerbated by the neoliberal ‘responsibilisation’ for one’s health, which widely ignores social determinants of health – including factors such as poverty, inequality, poor built environment, social exclusion, and poor public policies and services – that create and perpetuate health inequalities, and lead to compromised access and utilisation of healthcare services by people with disabilities [14]. Furthermore, this responsibilisation often becomes internalised; needing help is turned into a problem, associated with dependency and ensuing lost productivity, both for the person needing help and for the person providing it [33].

## The cases of CHILE and GREECE

### Chile

The 2015 Second National Study on Disability revealed that about 20% of Chileans have one or more disabilities, with more adult women than men having a disability (26% vs. 15%, respectively) [34]. A person with disability was defined as a person that has a physical, mental, or sensory impairment whose full participation in society is restricted [35]; this is the official definition of disability in the country, and it is the one used in all surveys and reports.

The limited research on disability and healthcare services in Chile has showed that people with disabilities face higher barriers in their access and utilisation of services, not only on account of their higher health needs but also due to structural disadvantage – such as unemployment, lower education, and lower incomes – that prevent them from accessing good-quality private healthcare coverage [15]; this is the result of what Schrecker [36] called ‘neoliberal epidemics’, that is the material effects of neoliberalism.

The organisation of the healthcare system in Chile is a legacy of the neoliberal policies that were undertaken during the military government of Augusto Pinochet (1973–1990). Such reforms extended to many areas, including the health system, pension system, education, and several state industries, and resulted in a reorientation of social policies and the de facto ending of the welfare state [37]. With regards to the healthcare system, a major milestone was the creation in 1981 of private health insurance institutions (ISAPREs, from their initials in Spanish). This separation of the health system between mainly FONASA (public healthcare provider) and ISAPREs constituted a “*regressive form of targeting [that] helped to deepen the crisis in the public health system*” ([38], p. 202). It also led to the formation of health inequalities, since it created stratification of healthcare services [14].

The real winners of the neoliberal healthcare reforms in Chile have been transnational companies, for instance insurance firms than manage the ISAPREs [39]. On the one hand, despite the slowing down of the Chilean economy in the last few years and the poor performance of other sectors – mainly mining and construction – the profits of the six main ISAPREs reached about USD 80 millions in 2016, an increase of 62.2% from the previous year [40]. This is due to the real power and influence that ISAPREs and the transnational companies behind them have on the Chilean economy and society, as seen by the recent revelations of significant donations from powerful business entities/conglomerates [41]. On the other hand, transnational companies that control many of the ISAPREs are based outside Chile, thus hampering government’s efforts to regulate ISAPREs. As Rotarou and Sakellariou [14] state,

“*while the privatisation of more health care services requires a greater vigilance of the private sector from the part of the government, weakened governments – under the neoliberal policies of promoting privatisation and cutting back on the capacity of the public sector – are less able to protect their members from abuses by third-party institutions*” (p. 500).

Chile has universal health coverage, in the sense that all citizens have access to healthcare services. FONASA is divided into four segments (A, B, C, and D), depending on individual or family income: people with very little or no income are affiliated with FONASA segment A, and access services free of charge. In 2014, the vast majority of Chileans (75.2%) were affiliated with FONASA, while 18.5% with an ISAPRE; the rest of Chileans either paid out-of-pocket for treatment and medicine or were affiliated with the health provider of the Armed Forces [42]. The stratification of the healthcare services can be seen by the fact that most people belonging to higher socioeconomic classes access the good-quality services and timely attention provided by the ISAPREs, while people from lower socioeconomic groups are affiliated with FONASA, which is characterised by poorer infrastructure and longer waiting times.

An analysis of data from the most recent National Socioeconomic Survey (CASEN) of the Government of Chile showed that in 2015, 23.3% of people with disabilities belonged to the lowest income quintile, as opposed to 19.7% of people without disabilities; on the other hand, only 13.8% of people with disabilities belonged to the highest quintile vs. 20.6% of people without disabilities [43]. Research [44] has found a strong correlation between socioeconomic indicators, such as income, and the likelihood of having a disability.

Another consequence of the adoption of neoliberal policies in the Chilean health system is the introduction of scaled contract premiums charged by ISAPREs, according to people’s age, sex, and perceived health risk. People with disabilities (together with other parts of the population, including women of reproductive age, and older people) are often excluded by ISAPREs that charge higher premiums to these groups than to single, healthy males [45]. As a result, in 2015 only 11.7% of the total ISAPRE affiliates were people over 60 years of age, only about 35% were women [46], and just 4% were people with disabilities [34]. Added to this, Araya et al. [47] have revealed that people with mental health needs are often excluded from ISAPREs due to cost, and are, therefore, forced to access the overburdened and underfunded FONASA.

Recent studies have also exposed deep inequalities in access to healthcare between people with and without disabilities in Chile. People with disabilities report increased barriers to accessing healthcare, stopping them from getting the care they need [15]; women with disabilities, for example, report lower use of cancer screening services compared to non-disabled women [48].

In an effort to increase equity in the healthcare system, a healthcare reform was initiated in 2000. One of the measures adopted included the establishment of the Universal Access with Explicit Guarantees (AUGE-GES) healthcare plan. The AUGE-GES guarantees the treatment and rehabilitation of all Chileans suffering from specific diseases that pose the greatest health impact to the population, independent of their ability to pay for such services and treatment [49]. Despite the benefits that the AUGE-GES plan has brought to the population, it has also had some unintended effects. As Martinez-Gutierrez and Cuadrado [50] point out, this reform created a new private market focussing on the conditions covered by the AUGE-GES plan, leading to a 329% increase in cash transfers from the public fund to private. Furthermore, out-of-pocket payments continue to be high, and rising, with the main driver being pharmaceuticals [50, 51], a particularly important issue for people with disabilities who usually need medicines on a regular and long-term basis.

### Greece

Since 2009, Greece has been dealing with the effects of the financial crisis and the subsequent recession [52]. The sovereign debt crisis that hit Greece resulted in the end of the Greek socioeconomic model of 1994–2008, and the transition from a mainly state-led familistic model characterised by heavy public indebtedness to a liberal, partly de-familialised capitalism [19]. The structural adjustment programmes / bailouts that Greece had to accept aimed at a significant reduction of public spending, and were guided by the neoliberal principles of deregulation, stabilisation, and privatisation [52]. These programmes have had serious negative socioeconomic impacts: extreme poverty reached 15% of the total population in 2015, general unemployment is at 23%, and pensions have been drastically cut [53, 54]. The most recent (2011) data on unemployment for disabled people in Greece show that only 15.5% of people that had a work limitation due to long-standing health problem and/or a basic activity difficulty were employed [55].

Unemployment and low income can lead to compromised access to healthcare, due to, for example, difficulties in paying for medication [56]. However, currently the public healthcare sector experiences severe shortages, leading to high waiting times and deterioration of services [56, 57]. Combined with what Adam and Papatheodorou ([58], p.271) called the “*dismantling [of] the feeble social protection system*”, these changes have left many people with limited support to respond to health risks.

Policies targeting specifically the health sector have had well-documented consequences on access to healthcare in Greece [59–62], including a ‘roll-back’ of health and social protection spending, and a “‘*roll-out’ of neoliberalism*” ([8], p. 313). Many vital health services, such as cancer-screening programmes, mental health services, and municipal public health services, have been severely cut [63]. Other measures have included increased admission fees and co-payments for medications, outpatient and diagnostic services in public hospitals [64, 65], mergers of hospitals and clinics [13], firing of 25,000 health staff, and a reduction in healthcare coverage and related healthcare benefits [66]. The situation might be further exacerbated since the 2017 state budget includes a cut of 200 million euros to state contribution to the national healthcare system [67].

Certain population groups defined as vulnerable have still access to free medications. These groups include disabled people in residential care or requiring hospitalisation or continued medication who are deemed as having over 67% impairment [68, 69]. The majority of disabled people, however, need to pay a 25% contribution for medications, like the rest of the population [70].

Writing to the Prime Minister of Greece, the National Confederation of Disabled People [71] outlined the impact of neoliberal, austerity-driven policies on access to healthcare for people with disabilities. Increased co-payment for medications is an example of a policy that while it applies to the entire population, it may affect disproportionately people with disabilities [15], since they may need medication on a regular basis. People with disabilities are also affected through reforms that specifically target them. For example, Law 4387 [72] stipulates a reduction of national pension proportionate to the severity of impairment, so that people who are deemed as being less able to work will eventually receive a reduced pension. By leading to a lower income, the outcome of this policy may indirectly affect access to healthcare for disabled people, who already suffer from structural disadvantage.

Greece recently implemented a health voucher scheme, which facilitates access to healthcare for uninsured people by allowing three visits to primary care within a four-month period [59]. This scheme, however, has not been very successful [64]. Reasons may include inadequate information about the scheme. More importantly, such measures need to take into account the complex nature of access to healthcare. Having free access to healthcare through such schemes will not benefit people who cannot even get to a healthcare facility because of, for example, lack of appropriate and affordable transportation [16].

## Disabled people and access to healthcare services: A discussion of pathways

Meade, Mahmoudi and Lee [73] developed the Model of Healthcare Disparities and Disability, to foreground the intersections between contextual and personal factors, and healthcare disparities. Broad contextual issues, such as transportation, policies, and health systems can have a big impact on how people with disabilities access healthcare. These are exactly the issues that are targeted by neoliberal policies, which by adopting free market values, do not address structural disadvantage experienced by groups of the population, such as disabled people. The ideals of individual self-reliance and responsibility are heavily promoted by neoliberalism, with members of disadvantaged groups – including people with disabilities – that rely on state support being considered ‘…*defective by reason of their financial dependence*’ ([74], p. 158), and unemployment seen as an individual failure. However, this neoliberal, decontextualised evaluation of people with disabilities completely ignores the extra challenges that this subgroup faces, not only with regards to compromised access to healthcare services, but also due to existing and perpetuated structural disadvantage.

Kentikelenis ([52], p.1) identified three main pathways through which neoliberal reforms, such as the ones that Chile went through and Greece is currently carrying out, affect access to healthcare. These are “*policies directly targeting health systems; policies indirectly impacting health systems; and policies affecting the social determinants of health*”. These policies can lead to a higher rate of unmet health needs, increased barriers in accessing healthcare, including preventive services, and a worsening of several health indicators [8, 52]. While policies such as budget cuts in the healthcare sector, a liberalised labour market, and reduction of unemployment benefits affect all parts of the population, people with disabilities might be disproportionately affected. Neoliberal, austerity-driven reforms “*may translate into the removal of hitherto available healthcare services, and reliance on expensive services provided by the private sector*” ([52], p.7), leading to catastrophic out-of-pocket payments, as has already been observed in Chile [51].

While these pathways appear to be applicable to the population as a whole, they do not draw attention to the specific issues faced by disabled people. Based on the case studies we presented, we propose that the pathways that affect access to healthcare for this population can be summarised as:

1. Policies directly or indirectly targeting healthcare, affecting the entire population, including disabled people (often disproportionately), for example, co-payments, budget cuts in health, etc.; and
2. Policies affecting socioeconomic determinants, directly or indirectly targeting disabled people, indirectly impacting access to healthcare, for instance, changes in the benefits system (see Table 1).

**Table 1:** Pathways through which neoliberal reforms affect disabled people’s access to healthcare services

Pathway	Examples	Effects for disabled people
Policies directly targeting healthcare	Budget cuts in the health sector; increased co-payments; reduced staff; increased privatisation.	Exclusion from services because of cost; higher unmet needs because of high waiting times and lack of staff; higher mortality and morbidity due to deterioration of healthcare services.
Policies indirectly targeting healthcare	Austerity reforms in the broader public sector, leading to hiring freezes; capital controls.	Higher unmet needs because of high waiting times; shortages in medications.
Policies affecting socioeconomic determinants, directly targeting disabled people	Changes in the benefits system; association of state pension with disability level.	Higher rates of poverty; less protection against poverty, unemployment, and healthcare risks; social exclusion.
Policies affecting socioeconomic determinants, indirectly targeting disabled people	Austerity-driven financial policies leading to an increase in unemployment and poverty; reduced labour costs.	Higher rates of poverty; less protection against poverty, unemployment, and healthcare risks; social exclusion; increased prevalence of mental illness.

The case studies from Chile and Greece evidence some of the ways that new configurations of the state, exemplified through neoliberal reforms, can affect access to healthcare for people with disabilities. This population is affected by reforms, that either impact directly healthcare, such as the privatisation of healthcare, the outsourcing of services, increased costs for access to healthcare and medications, or indirectly through budget cuts in the state sector. Often needing more healthcare services (including medications), experiencing high unemployment, and belonging to lower income quintiles, people with disabilities are particularly disadvantaged by such neoliberal policies. Furthermore, living with a disability implies extra costs (for instance, additional medication costs or heating costs for physically-disabled people that stay more at home in winter), which further disadvantages this population [75].

Neoliberal reforms can be seen as a form of structural violence, disproportionately affecting the most vulnerable parts of the population, curtailing, directly and indirectly, access to basic rights, such as healthcare. Sparke ([76], p.287) argues that neoliberal restructuring of states leads to a “*biological sub-citizenship*”, where people embody in the form of ill health the effects of neoliberalism. The power differentials produced, reproduced, and exacerbated through neoliberal reforms that focus on economic rather than human rights indicators, lead to a category of disempowered people, subordinated to the markets. The examples of Chile and Greece illustrate the effects this subordination can produce, which range from catastrophic out-of-pocket payments to compromised access to healthcare.

## Final comments

Neoliberal policies have led to an individualisation of the right to health and a reconceptualisation of “*health care [...] as a private good for sale rather than a public good paid for with tax dollars*” ([10], p. 84) This relocation of care from the welfare state to the free market has a detrimental effect on access to healthcare services for groups that are already experiencing difficulties, such as people with disabilities. Evidence from Chile [14] shows that neoliberal reforms have produced long-lasting, negative effects on health, disproportionately affecting the most vulnerable parts of the population. In the case of Greece, the recent austerity-driven policies not only have they had a negative impact on the health of the general population and the quality of healthcare services, but they have also led to increased insecurity for disabled people, as they often need to co-pay for their medication while in need of medicine on a regular basis [16, 62, 70]. Overall, increased healthcare needs due to the presence of disability, combined with the negative effects of neoliberal policies and the structural disadvantage people with disabilities often face, can lead to increased barriers to healthcare access for this population.

Since health inequalities are usually aggravated by neoliberal policies, political commitment together with public understanding and support of measures reducing health inequalities are necessary in order to address both the increased healthcare needs of disabled people but also to tackle underlying health determinants. Several authors [77, 78], have called for the development of strong protection policies to address health inequalities in the current climate of neoliberalism-driven reforms observed in many countries. It is important to carry out health equity impact assessments to evaluate the effectiveness of any policy initiatives.

International evidence suggests that reducing health inequalities can be achieved by targeting low socioeconomic position of people with disabilities (for example, by improving education, and increasing employment opportunities), improving intermediary factors (for instance, improving health behaviours, accessing good nutrition, providing access to good-quality healthcare services, creating healthy living and working conditions), and reducing the impact of ill health on socioeconomic status (for example, through disability benefits and reintegration programmes) [79, 80]. The effectiveness of these measures in improving access to healthcare for people with disabilities needs to be evaluated. Overall, it is imperative that, particularly in times of austerity, special care and attention is paid to the needs of people with disabilities – especially with regards to their health– in order to promote their socioeconomic inclusion and protect their human rights.

## Abbreviations

AUGE-GES: Universal Access with Explicit Guarantees [Acceso Universal a Garantías Explícitas en Salud]
CASEN: National Socioeconomic Survey [Encuesta de Caracterización Socioeconómica Nacional]
DPM: Disabled People’s Movement
FONASA: Public Healthcare Provider [Fondo Nacional de Salud]
ISAPREs: Private Health Insurance Institutions [Las Instituciones de Salud Previsional]
OECD: Organisation of Economic Development and Cooperation
WHO: World Health Organisation
